# fNIRS Hyperscanning of Negotiation: The Role of Personality and Neural Synchronization

**DOI:** 10.3390/brainsci16060601

**Published:** 2026-05-31

**Authors:** Michela Balconi, Angelica Daffinà, Laura Angioletti, Federica Saquella, Carlotta Acconito

**Affiliations:** 1 International Research Center for Cognitive Applied Neuroscience (IrcCAN), Università Cattolica del Sacro Cuore, 20123 Milan, Italy; 2 Research Unit in Affective and Social Neuroscience, Department of Psychology, Università Cattolica del Sacro Cuore, 20123 Milan, Italy; 3 Faculty of Medicine, Università degli Studi di Milano, 20122 Milan, Italy

**Keywords:** fNIRS, hyperscanning, personality traits, negotiation, decision-making

## Abstract

**Highlights:**

**What are the main findings?**

- Neural synchronization differed during naturalistic negotiation, reflecting differences in cognitive processing between interacting individuals.
- Decision-making style and personality traits modulate brain activity during negotiation.

**What are the implications of the main findings?**

- Neural synchronization during negotiation is shaped by individual characteristics.
- Integrating neural and psychological measures may inform future research on social interaction and negotiation strategies.

**Abstract:**

Background: Decision-making is often a shared and collective process, facilitated by negotiation dynamics. This study adopted fNIRS hyperscanning to explore similar brain hemodynamic responses during naturalistic negotiation and decision-making interactions and the role of individual differences. Methods: Homologous dyads of speaker A and speaker B engaged in a realistic negotiation task, deciding how to handle a non-conforming team member. The task included three steps: the Individual step (Indstep), where the participants individually selected how to decide; the Cooperation step (Coopstep), involving collaborative negotiation; and the Agreement step (Agrstep), where a mutual agreement was reached. General Decision-Making Style (GDMS), Maximization Scale (MS) and Big Five Inventory (BFI-10) were also administered. Results: Higher deoxygenated hemoglobin (HHb) dissimilarity emerged when speaker B was speaking and speaker A was listening, suggesting that the members exhibited differences in the level of cognitive demand required for the conversation, or that speaker A was attempting to assert his perspective. Moreover, the avoidant decision-making style, the alternative-search tendency, and the decision-making difficulties subscales negatively correlated with HHb dissimilarity in the left hemisphere during the Agrstep, as well as the extraversion trait during the Indstep and the Coopstep, highlighting how individual differences modulate the neural mechanisms underlying negotiation. Conclusions: These findings reveal that brain patterns of neural similarity in negotiation contexts are sensitive to both conversational roles and individual decision-making profiles. Integrating a hyperscanning paradigm with psychological assessment offers interesting insights into the neurocognitive foundations of cooperation, revealing the influence of cognitive and personality traits on neural activity associated with the naturalistic negotiation process.

## 1. Introduction

Decisions are rarely made in isolation, as individuals are embedded in social networks where others’ choices influence their own through cognitive mechanisms like theory of mind and social cognition, with negotiation dynamics facilitating joint action [1,2,3]. Negotiation can be defined as a social process in which individuals with partially different preferences attempt to reach a shared decision by coordinating their goals, exchanging perspectives, and mutually adjusting their positions. In this context, the success of negotiation is not solely dependent on individual outcomes, but also on the ability to achieve joint solutions that satisfy both parties and promote cooperative interaction [4,5]. In naturalistic interactions, this process typically unfolds across three broad phases: an initial articulation of individual preferences and positions, an interactive exchange phase in which perspectives are expressed, discussed, and progressively aligned, and a final integrative phase in which a joint decision is established [6,7].

When exploring negotiation, it is essential to consider individual characteristics that may impact the process [2], such as (i) personal background (e.g., sex, culture); (ii) abilities (e.g., intelligence, creativity); (iii) personality traits (e.g., extroversion, decision-making styles); (iv) motivations (e.g., prosociality); and (v) expectations and beliefs (e.g., self-efficacy). Previous studies on decision-making and cooperative behaviour have highlighted how individual differences in decision-making styles, personality traits, and interpersonal orientation may influence negotiation dynamics [8,9,10,11,12]. Despite the high availability of validated tools to assess individual differences and the awareness of their relationship to the outcomes of decision-making and negotiation processes, there has been little emphasis on dyadic relationships [13], especially those explored from a neuroscientific perspective.

Indeed, from a neuroscientific perspective, investigating interpersonal brain neural coupling among individuals engaged in social interactions, such as negotiation, offers valuable insights [14,15,16], moving beyond the constraints of a single-brain approach. Adopting the second-person approach alongside the hyperscanning paradigm would enable the exploration of the brain synchronization of two or more interacting individuals, considering them interconnected components of a dynamic system, constantly influencing and adapting to each other [17,18,19]. Specifically, according to the literature [20], the level of brain synchronization could be investigated in terms of dissimilarity index (calculating the Euclidean distance index—EDi), quantifying the extent to which, within each dyad, brain activity differs among the individuals.

Among the neuroscientific tools used in the hyperscanning paradigm, functional Near-Infrared Spectroscopy (fNIRS) is widely used to study social cooperative interactions [16,17,21,22]. This technique monitors brain activity by tracking infrared light through the cortex and offers high spatial resolution while being less affected by motion or noisy environments, overcoming some of the limitations of electroencephalography (EEG) and Magnetic Resonance Imaging (fMRI) [23]. Brain activation increases regional cerebral blood flow (rCBF), leading to an increase in oxygenated hemoglobin (O2Hb) and a decrease in deoxygenated hemoglobin (HHb), both markers of neural activity [24]. Conversely, increased HHb and decreased O2Hb levels are linked to neural inhibition, indicating reduced neural activity or the suppression of specific brain regions during task engagement [25]. Although O2Hb is more commonly analyzed due to its higher sensitivity and signal-to-noise ratio [26], HHb also offers valuable insight into activation and inhibition mechanisms.

Previous fNIRS studies on cooperation through spoken language suggested that frontal regions play a key role in successful knowledge sharing [27], as they integrate message relevance into self-concept, aiding in effective interaction and agreement [28,29]. Despite these interesting findings, to the best of our knowledge, no previous studies have explored the negotiation process adopting a fNIRS hyperscanning approach.

Alongside the functional meaning attributed to O2Hb and HHb, different studies explored the lateralization effect. An interesting interpretative perspective emerges from the studies of Killgore and Yurgelun-Todd [30], who propose a distinction between emotional and cognitive processing: the right hemisphere would be responsible for the processing of emotional information—especially non-verbal signals, facial expressions, and tone of voice—whereas the left hemisphere would be more involved in the management of cognitive information, such as language and analytical reasoning; this perspective considers a complex organization in which the right hemisphere deals with affective and intuitive components, while the left hemisphere focuses more on logical components.

Clinical studies support this perspective, highlighting that right-brain damage often reduces emotional expression through voice, gestures, and facial cues [31]; moreover, Vallar et al. [32] observed that the left hemisphere is dominant in decision-making tasks, regardless of content type. Recent studies on decision-making [33] also have interpreted the results of famous experiments such as the Asian Disease Problem [34] and the framing effect [35], suggesting that the left hemisphere is more inclined to separate context from relevant information to achieve an effective choice, while the right hemisphere may be more influenced by contextual factors.

While traditional studies on emotional and cognitive lateralization have primarily focused on individual brain processes, advances such as fNIRS hyperscanning enable the study of brain synchronization in more dynamic real-world interactions, where both hemispheric functions are integrated [36,37,38].

Building on these theoretical premises and addressing the gap in the literature, the present study adopted an fNIRS hyperscanning paradigm to explore patterns of neural similarity during a negotiation-based decision-making task in a naturalistic setting. This approach not only allows for the examination of prefrontal activation patterns linked to emotional and cognitive lateralization, but also provides insight into how personality traits influence cooperative behaviour. Furthermore, to assess individual differences in decision-making style and personality traits, self-report questionnaires (i.e., GDSM, MS, and BFI-10) were administered.

Concerning the hypotheses, since an effective negotiation process may be associated with more similar neural responses in the frontal area, a lower dissimilarity of O2Hb was expected in this area, as an indicator of neural activation and perspective integration. Specifically, this lower dissimilarity was hypothesized to vary across phases and speaking turns, reflecting greater adjustment and integration efforts.

Moreover, the question was raised as to whether individual characteristics (as a more cognitive or affective approach) that might affect the negotiation process were related to the lateralization of hemodynamic activity during the three phases of the process. Although no previous study investigated this relationship in negotiation, it was expected that it mainly involves left activation, since negotiation requires cognitive processes of perspective integration.

Additionally, based on the meaning of the different personality traits, it was expected that individual differences in decision-making tendencies and personality traits would modulate inter-brain neural dynamics during negotiation. Specifically, traits associated with social orientation (i.e., extraversion and agreeableness) should facilitate interpersonal coordination and be associated with lower inter-brain dissimilarity; conversely, decision-making difficulties and avoidant tendencies should be associated with higher inter-brain dissimilarity.

## 2. Materials and Methods

### 2.1. Sample

The present study involved 13 homologous dyads of student volunteers, 10 female and 3 male dyads (Age_mean_ = 25.42 years; Age_SD_ = 6.37 years), to reduce variability associated with gender-related differences in communicative, cooperative, and negotiation dynamics [39,40,41]. The participants were recruited using a non-probability convenience sampling method. An a priori power analysis was conducted using G*Power (version 3.1.9.7) [42] for a repeated-measures ANOVA design, assuming a medium-to-large effect size (f = 0.40), α = 0.05, power = 0.95. The estimated minimum sample size was 12 dyads.

Prior to the beginning of the experiment, the participants were randomly assigned to one of two experimental roles, labelled speaker A and speaker B, thus creating 13 same-sex pairs. Strict control procedures were implemented to ensure that no prior knowledge existed between the members of each pair, thus minimizing the risk of bias or confounding variables that could influence the experimental results. All the participants were right-handed and had normal or corrected vision. In addition, subjects with a history of psychiatric or neurological disorders, significant depressive symptoms or impaired cognitive function, as well as participants with short- or long-term memory deficits or taking psychoactive drugs were excluded.

The present study was conducted in accordance with the ethical principles outlined in the Declaration of Helsinki (2013) and followed the General Data Protection Regulation (GDPR, EU Reg. 2016/679) [43]. Ethical approval was provided by the Ethics Committee of the Department of Psychology, Catholic University of the Sacred Heart, Milan, Italy.

### 2.2. Experimental Procedure

The study was conducted in a moderately illuminated room where participants were seated close to each other to facilitate face-to-face interaction and minimize external interference. Before the beginning of the experiment, all the participants gave their informed consent and were provided with comprehensive instructions to ensure clarity and compliance with the study guidelines. Moreover, the participants were instructed to maintain calm, clearly articulate their speech (avoiding whispering), and take turns to ensure that the dialogue did not overlap. To prevent any influence of non-verbal cues on the outcome, video recordings were also used to monitor any significant changes in facial expressions or body posture. The neural activity of the dyads was recorded using an fNIRS hyperscanning paradigm, collecting data during both the 120 s baseline and the main experiment.

**Figure 1:**
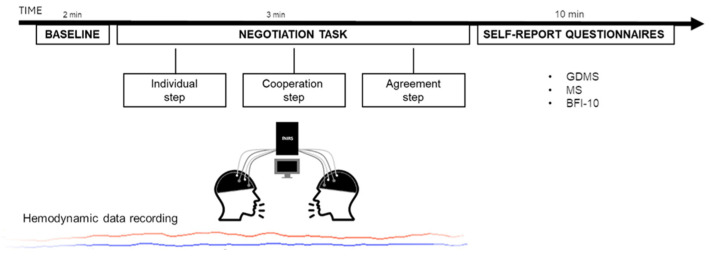
The experimental procedure. A graphical representation of the entire procedure, with baseline, negotiation task with the three different steps, and self-report questionnaires.

The experiment procedure, which lasted approximately 30 min, included a negotiation task and the administration of self-report questionnaires to explore individual traits (Figure 1).

### 2.3. Negotiation-Based Decision-Making Task

In the negotiation-based decision-making task, the participants were engaged in a collaborative communicative task designed to solve the following realistic scenario: “*A member of your group is not conforming to the group’s values and working style. How would your real group handle this situation*?”

Specifically, this phase was organized into three steps: (i) the Individual step (Indstep), (ii) the Cooperation step (Coopstep) and (iii) the Agreement step (Agrstep). The Individual step (Indstep) represented the first initial part of the decision-making negotiation. Here, the participants individually selected one of eight statements describing potential strategies their real group might adopt in response to the scenario (i.e., “*The group discusses the issue collectively, allowing all members to share their opinions before reaching a collective decision*”; “*The decision is made based on the preference of the majority of the group*”; “*The decision is guided by the perspective of the most experienced member of the group*”). Then, the subjects were invited to articulate their choice to the other members of the dyad, while the experimenter recorded their responses, denominating them as Initial choice.

In the Cooperation step (Coopstep), the participants were given time to discuss and collaboratively select a single statement that best represented their shared agreement from the eight options previously presented. This step involved a collaborative process where the dyad negotiated, compared, and adjusted their initial choices to reach a consensus.

In the final step, the Agreement step (Agrstep), the participants achieved a shared agreement and articulated their consensus, thereby representing the collective decision of the dyad. This step marked the convergence of individual preferences into a unified outcome. The experimenter documented the outcome of this negotiation as the final choice, reflecting the collective agreement.

#### Self-Report Questionnaires

At the end of the negotiation-based decision-making task, the participants were invited to complete the Italian versions of the following questionnaires to collect individual differences: the General Decision-Making Style (GDMS) [44,45], the Maximization Scale (MS) [46,47] and the 10-item Big Five Inventory (BFI-10) scales [48].

Specifically, the GDMS is a 25-item questionnaire rated on a 5-point Likert scale, to assess five decision-making styles: rational, intuitive, dependent, avoidant and spontaneous. Rational individuals carefully analyze options, while intuitive individuals rely on gut feelings and patterns. Dependent individuals often seek advice from others, avoidant individuals tend to avoid making decisions, and spontaneous individuals make quick, unhesitating decisions.

Concerning the MS, it consists of 13 items on a 7-point Likert scale and is used to assess decision-making styles. It distinguishes between maximization, the tendency to optimize decisions through extensive information-seeking and social comparison; and satisfaction, the tendency to settle for a “good enough” option. It also includes three subscales: high standards, alternative-search, and decision-making difficulties.

Finally, the BFI-10 is a brief version of the Big Five Inventory, composed of 10 statements on a 5-point Likert scale, designed to assess the five major personality traits: openness, which reflects a willingness to engage in new experiences; conscientiousness, indicating a tendency to be organized and responsible; extraversion, related to an individual’s interest in the external world; agreeableness, representing the inclination to act cooperatively and empathetically; and emotional stability, which refers to being calm, stable, and equilibrated.

Furthermore, the participants were asked to complete an additional exploratory self-report questionnaire assessing their subjective experience of the interaction, including perceived influence, trust, cooperation, mutual respect, and tension during the negotiation process.

### 2.4. fNIRS Data: Acquisition and Processing

**Figure 2:**
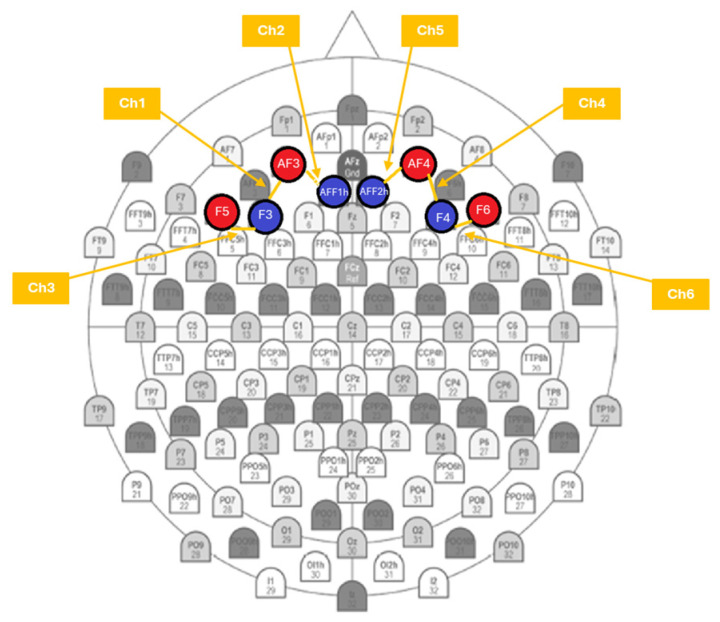
The fNIRS setup. The four light sources (AF3, F5, AF4, and F6) are indicated by red markers, while the four detectors (F3, AFF1h, AFF2h, and F4) are indicated by blue markers. The six channels are represented by yellow lines: Ch1 (AF3-F3), Ch2 (AF3-AFF1h), Ch3 (F5-F3), Ch4 (AF4-F4), Ch5 (AF4-AFF2h), and Ch6 (F6-F4).

The NIRScout system (NIRx Medical Technologies, LLC, Los Angeles, CA, USA) was adopted to assess changes in oxygenated (O2Hb) and deoxygenated hemoglobin (HHb) levels during the baseline conditions and the negotiation phase. The positions of four light sources (AF3, AF4, F5 and F6) and four detectors (AFF1h, AFF2h, F3 and F4) on the subject’s scalp were defined according to the international 10/20 system [48]. The optodes, which emitted near-infrared light at two wavelengths (760 and 850 nm), were positioned using an fNIRS headset. The distance between each source–detector pair was measured to be 30 mm and covered the frontal areas of the brain, as indicated by the Automated Anatomical Labeling atlas and the Brodmann area system [49,50], with the support of the fOLD software v2.2 (fNIRS Optodes’ Location Decider) [51]. This arrangement resulted in six channels: Ch1 (AF3-F3), Ch2 (AF3-AFF1h), Ch3 (F5-F3), Ch4 (AF4-F4), Ch5 (AF4-AFF2h) and Ch6 (F6-F4). The data were acquired at a sampling rate of 6.25 Hz using NIRStar software version 12.4 (NIRx Medical Technologies LLC, Glen Head, NY, USA) (Figure 2).

Following this, the data were processed with nirsLAB software (v.2014.05; NIRx Medical Technologies LLC, Glen Head, NY, USA) and a digital band-pass filter (0.01–0.3 Hz) was applied to convert the data offline into mmol*mm values, thus reflecting changes in O2Hb and HHb. Any artefacts caused by eye movements, muscle contractions or body movements were then visually examined. Channels with insufficient optical coupling or lacking the typical heartbeat oscillations (~1 Hz) were excluded [52]. To further reduce the respiratory noise (~0.3 Hz), a linear-phase FIR filter was applied, which ensures a symmetrical impulse response [53,54]. Statistical analysis was conducted by calculating the effect size (Cohen’s d) for each channel, according to the formula (m2 − m1)/s, where m1 and m2 represent the mean values of the concentration level during the baseline and the negotiation interaction, while s is the standard deviation of the baseline. To improve the signal-to-noise ratio, effect sizes were averaged across the six channels. Given that the raw fNIRS data were relative and did not allow for a direct comparison between participants or channels, the normalized effect size values were combined without considering the differential pathlength factor (DPF), which did not affect the effect size calculations.

### 2.5. Data Analysis

Before conducting statistical analyses, skewness and kurtosis tests were conducted to verify the data’s normality. Then, two different statistical analyses were performed: fNIRS dyadic data analysis and correlation analysis between the self-report and fNIRS data.

#### 2.5.1. fNIRS Dyadic Data Analysis

For the analysis of the fNIRS data, speaking turns related to the entire negotiation steps were considered; thus, the interaction between participants was analyzed considering entire specific moments in which one participant spoke and the other one listened, alternating these roles. In doing so, two conditions were defined: a first condition, designated “A-Speaking condition”, considered all the moments in which speaker A spoke and speaker B listened; and a second condition, designated “B-Speaking condition”, considered all the moments in which speaker B spoke and speaker A listened. This approach enabled the analysis of the moments during which both participants could explain their opinions and negotiate them. Speech onsets and offsets were identified through the analysis of the video recordings and verbatim transcriptions of the interaction. Three trained research psychologists independently coded the dyadic interactions and identified the relevant moments of speaking and listening turns. Moreover, it is important to note that periods of conversational pauses and expressions not directly connected to the negotiation process were excluded from the analysis. Data segments for each participant within the dyad were defined based on the average duration of speech instances in which they were actively engaged in negotiation communication.

To explore patterns of neural similarity, the degree of difference between the individuals’ O2Hb and HHb levels within each dyad was determined by calculating the O2Hb and HHb Euclidean distance index (O2Hb-EDi and HHb-EDi). Two Regions of Interest (ROIs) were considered concerning brain lateralization for the statistical analysis. In particular, the left side included Ch1 (AF3-F3), Ch2 (AF3-AFF1h), and Ch3 (F5-F3), while the right side included Ch4 (AF4-F4), Ch5 (AF4-AFF2h) and Ch6 (F6-F4).

Thus, for the fNIRS data, two repeated-measures ANOVAs with Condition (2: A-Speaking, B-Speaking), Lateralization (2: Left, Right) and Step (3: Indstep, Coopstep, Agrstep) as within-subject factors were performed on O2Hb-EDi and HHb-EDi, as distinct dependent variables. The degrees of freedom of the ANOVA test were adjusted using the Greenhouse–Geisser epsilon when necessary to account for violations of sphericity. Significant interactions were further explored through pairwise comparisons to evaluate simple effects, while the Bonferroni correction was applied to control for potential biases resulting from multiple comparisons. The size of statistically significant effects was assessed using eta squared (η^2^) indices, with a significance level of α = 0.05.

#### 2.5.2. Correlation Analysis Between Self-Report and fNIRS Data

To investigate whether individual differences were linked to the lateralization of the hemodynamic activity during the three different steps of the negotiation phase, the EDi was calculated for each set of self-report data and relative subscales. Specifically, for each dyad, the EDi was computed as the absolute difference between speaker A and speaker B scores for each questionnaire subscale, thus reflecting the degree of intra-dyadic dissimilarity in personality traits and decision-making styles.

Moreover, Pearson correlation analyses were performed between HHb-EDi and the EDi subscale scores from the self-report measures (GDMS, MS and BFI-10). Bonferroni correction was applied to control for multiple comparisons.

## 3. Results

**Figure 3:**
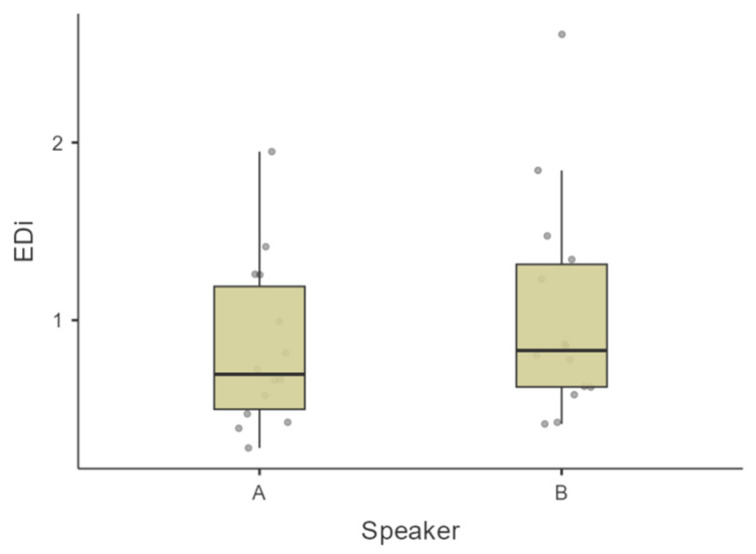
The fNIRS results. The boxplots display the distribution of HHb values for the two speaker conditions (speaker A and speaker B). The plots illustrate the median, interquartile range, variability, and potential outliers for each condition. Higher HHb values were observed in the speaker B condition compared to speaker A.

For the fNIRS data, the ANOVA reported a significant main effect on Condition (F [1,12] = 9.241, *p* = 0.010, ƞ2 = 0.012), with a higher level of HHb dissimilarity for the B-Speaking condition (high HHb-EDi) compared to the A-Speaking condition (low HHb-EDi) (Figure 3).

No other results for HHb and no results for O2Hb were found.

**Figure 4:**
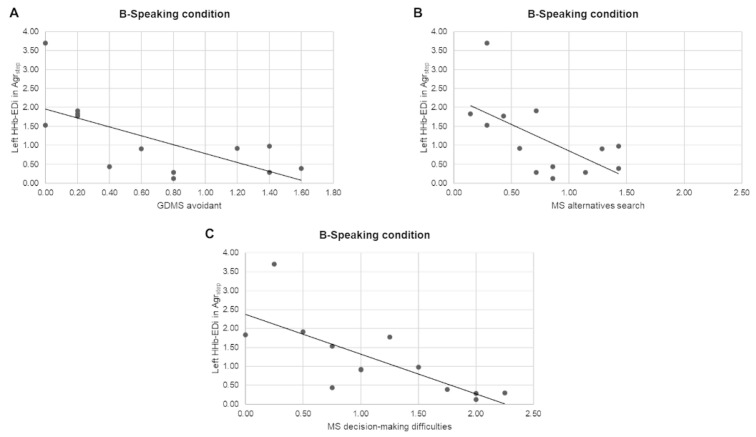
The correlation results between GDMS, MS and fNIRS. Scatterplots for statistically significant correlations between: (**A**) GDMS avoidant-EDi and HHb-EDi in the left hemisphere during the Agrstep in the B-Speaking condition; (**B**) MS alternative-search subscale-EDi and HHb-EDi in the left hemisphere during the Agrstep in the B-Speaking condition; (**C**) MS decision-making difficulties subscale-EDi and HHb-EDi in the left hemisphere during the Agrstep in the B-Speaking condition. Each point represents one dyad. The black straight lines represent the global linear trends.

Correlation analyses performed between the self-report and fNIRS data reported for the GDMS a negative correlation between GDMS_avoidant-EDi and HHb-EDi in the left hemisphere during the Agrstep in the B-Speaking condition (r = −0.671, *p* = 0.012) (Figure 4A).

Moreover, regarding the MS questionnaire, negative correlations were found between MS_alternatives-search subscale-EDi and HHb-EDi in the left hemisphere during the Agrstep in the B-Speaking condition (r = −0.615, *p* = 0.025) (Figure 4B) as well as between MS_decision-making difficulties subscale-EDi and HHb-EDi in the left hemisphere during the Agrstep in the B-Speaking condition (r = −0.725, *p* = 0.003) (Figure 4C).

**Figure 5:**
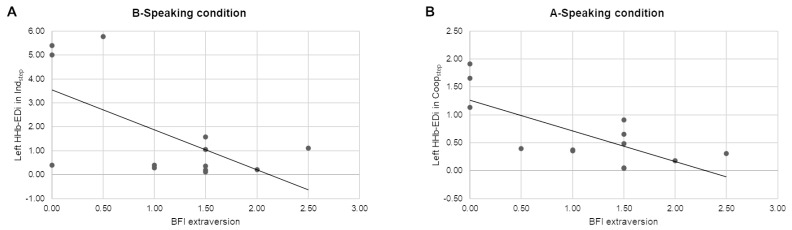
The correlation results between BFI-10 and fNIRS. Scatterplots for statistically significant correlations between: (**A**) BFI-10 extraversion and HHb-EDi in the left hemisphere during the Indstep in the B-Speaking condition; and (**B**) BFI-10 extraversion HHb-EDi in the left hemisphere during the Coopstep in the A-Speaking condition. Each point represents one dyad. The black straight lines represent the global linear trends.

Finally, for the BFI−10, negative correlations were found between BFI_extraversion and HHb-EDi in the left hemisphere during the Indstep in the B-Speaking condition (r = −0.616, *p* = 0.025) (Figure 5A) and between this personality trait and HHb-EDi in the left hemisphere during the Coopstep in the A-Speaking condition (r = −0.737; *p* = 0.004) (Figure 5B).

Post-task self-report questionnaires, designed to assess the participants’ subjective experience of the interaction (including perceived influence, trust, cooperation, mutual respect and tension), were also briefly described descriptively. Overall, these ratings indicated positive interaction experiences characterized by high levels of cooperation (M = 4.29, SD = 0.73), mutual respect (M = 4.42, SD = 0.64), trust (M = 4.21, SD = 0.71), and levels of interpersonal tension (M = 1.54, SD = 0.91), during the negotiations.

## 4. Discussion

This study used an fNIRS hyperscanning paradigm to explore patterns of neural similarity and the influence of personality traits on neural activity during a naturalistic negotiation and decision-making interaction. Specifically, avoidant decision-making, alternative-search tendency, decision-making difficulties, and extraversion were associated with lower HHb dissimilarity, highlighting the role of both interpersonal dynamics and individual differences in shaping the neural mechanisms underlying negotiation.

### 4.1. Neural Dissimilarity During Negotiation Speaking Turns

Dyadic analyses performed on the fNIRS data evidenced high HHb-EDi for the B-Speaking condition compared to the A-Speaking condition, suggesting greater dissimilarity when speaker B was speaking and speaker A was listening, compared to when speaker A spoke and speaker B listened. This finding is consistent with prior hyperscanning studies which demonstrate that the direction and quality of communication modulate interpersonal neural synchronization [19,55]. Since the HHb signal was previously associated with neural inhibition [25], this result indicates higher dissimilarity in the B-Speaking condition (when speaker B spoke and speaker A listened) suggesting reduced similarity in processing speaker B’s message within the HHb component [56,57,58,59]. These findings are consistent with prior fNIRS hyperscanning studies identifying the prefrontal cortex as a key region for inter-brain neural coupling during communicative and cooperative interactions [55,60] and extend this evidence to the specific context of naturalistic negotiation. Furthermore, the interpretation of these findings relies on the behavioural framework adopted in the present study. In this framework, video recordings were used to accurately segment speech turns and align conversational roles over time, while post-task self-report measures allowed us to describe the interaction context in terms of cooperation, trust, and low conflict. Conversely, lower HHb dissimilarity in the A-Speaking condition (when speaker A spoke and speaker B listened) suggested more similar neural responses, indicating a similar level of cognitive demand. Taken together, these findings suggest that when speaker B was speaking, the members exhibited differences in the level of cognitive demand required for the conversation. Indeed, one of the two members reported greater neural inhibition than the other, who probably needed to employ more cognitive resources to process the message, a process typically associated with the frontal region [16,28,29]. This could reflect a negotiation dynamic where one person tries to prevail over the other, advancing his or her idea. Additionally, another possible explanation may be attributed to the members’ personality traits, as individual differences impact both the negotiation process and its outcome [13,61,62]. Further studies should better explore these aspects, focusing on the different roles played by members during negotiation.

### 4.2. Individual Differences and Neural Dissimilarity During Negotiation

Focusing on the second analysis performed in this study, the correlation analyses indicated that higher dissimilarity in decision-making styles (GDMS), decision-making tendency (MS) and personality traits (BFI-10) correlated with lower HHb dissimilarity in the left hemisphere during the three different negotiation steps. It is important to highlight that all significant correlations were found in the left hemisphere, supporting the idea that negotiation is primarily cognitive [30], and consistent with fNIRS evidence of left-lateralized network engagement during interpersonal communication [55]. Also, the decision to focus solely on HHb was based on the significance observed in the previous analysis, which suggested that this hemodynamic index (rather than O2Hb) was specifically associated with the negotiation process, a choice supported by the previous literature documenting that significant effects may emerge selectively in one hemodynamic signal without a corresponding mirror effect in the other [57,58].

The negative correlation between the avoidant decision-making style and HHb levels in the left hemisphere during the Agrstep in the B-Speaking condition evidenced that when the two members differ more in their tendency to avoid decisions [46], their neural responses tend to become more similar, especially when they reach an agreement. Despite their different decision-making strategies, both the avoidant and non-avoidant members may exhibit a similar neural deactivation response in this step, probably due to a reduction in cognitive demands: the avoidant member may feel relieved, while the non-avoidant member may perceive the outcome as the successful completion of the negotiation. This interpretation could also be adopted to explain the two negative correlations found between the MS subscales and HHb levels in the left hemisphere during the Agrstep in the B-Speaking condition. Regarding the alternative-search (MS) correlation, it could be that the member who actively explored options has completed their evaluation, while the member preferring a predefined solution feels validated by the outcome [47]. Similarly, reaching an agreement (MS) may have reduced stress and cognitive effort for the member with high decision-making difficulties, as the shared decision marked the end of a stressful process. Likewise, for the member with low decision-making difficulties [47], this phase also brought relief as it signified the conclusion of an interaction that required focus and negotiation. Overall, these findings are in line with previous studies on collaborative decision-making, showing that neural convergence may emerge when interacting partners reach a shared outcome [63].

Finally, two negative correlations were found between the extroversion trait of BFI-10 and HHb levels in the left hemisphere during the Indstep in the B-Speaking condition and during the Coopstep in the A-Speaking condition. The similar cortical deactivation in Indstep suggests that this step did not require cognitive effort for the collaborative negotiation process, but only expressing one’s point of view. The extroverted member, comfortable with interaction [48], could express and share ideas without difficulty, while the introverted member might overcome his/her shyness, as the decision-making process is individual and does not require active confrontation with the other person, at least at this step. Instead, in the Coopstep, the members must adapt to the task of cooperating and finding common solutions. The introverted member, maybe more anxious or reluctant to participate actively [48], could adjust to the situation by finding ways of interacting that reduce pressure. Meanwhile, the extroverted member, more inclined to take a dominant role, begins to feel the need to adapt to the collaborative dialogue. Negotiation, therefore, is no longer an individual process but a mutual interaction where cognitive processing becomes more automatic and focused on reaching an agreement. This could lead to neural reduction, as the mind no longer faces internal cognitive conflicts but instead works in synchrony with the other member, decreasing the need for complex processing. This interpretation aligns with evidence that personality traits modulate both the behavioural and neural correlates of social engagement, and with accounts of neural alignment during cooperative interaction in which mutual adaptation leads to similar cortical responses [8,39].

## 5. Conclusions

To sum up, the findings of this fNIRS hyperscanning study suggested that the dyadic neural responses were more similar when speaker A was speaking and speaker B was listening, possibly indicating a lower neural dissimilarity associated with the cognitive resources required for message integration. Conversely, during the B-Speaking condition (when speaker B spoke, and speaker A listened), the greater neural dissimilarity suggested that the cognitive load was processed differently by each individual. Furthermore, individual differences, such as decision-making style, decision-making tendency and personality traits, impacted the neural responses during different phases of the negotiation. To the best of our knowledge, no other studies have examined neural dissimilarity across distinct phases of a structured negotiation paradigm while simultaneously accounting for individual differences in decision-making style and personality, providing a more comprehensive account of the neural mechanisms underlying dyadic negotiation than previously available.

Despite offering valuable insights, this study has several limitations. First, the present study focused exclusively on prefrontal cortical regions due to the adopted fNIRS montage, which limits the interpretation of the findings to prefrontal hemodynamic dynamics and does not allow inferences about whole-brain processes involved in negotiation. Secondly, the relatively small sample size and the exclusive use of university students limits generalizability, as students may differ from other groups, such as professionals, in cognitive, emotional, and social aspects that can affect negotiation. Future studies should include participants with varied backgrounds and experiences. Therefore, the present findings should be considered exploratory, requiring replication in larger samples. Additionally, relying solely on fNIRS technology, which measures only brain activity, restricts the scope of analysis. Further research with complementary methods, such as biofeedback, could help capture emotional components through autonomic responses. Also, the present study, focusing primarily on neural similarity and individual differences during negotiation processes, did not analyze non-verbal behavioural cues (e.g., gestures, facial expressions and other interactive signals), even though these were recorded. However, we acknowledge that non-verbal communication plays a significant role in natural interactions, and future studies could incorporate the analysis of non-verbal communication to offer a more thorough characterization of dyadic coordination. Furthermore, although the present paradigm requires all dyads to reach a final agreement, real-life negotiations do not necessarily end in consensus. Consequently, future studies may benefit from the adoption of more flexible paradigms, thereby enabling dyads to either reach or not reach an agreement within a defined duration, thus enhancing the ecological validity of the task. Finally, while personality traits were examined, other individual factors like personal history, motivations, and beliefs could also influence negotiation dynamics and should be considered in future research for a more comprehensive understanding of the factors influencing the negotiation process.

## Data Availability

The data presented in this study are available on request from the corresponding author due to ethical reasons for sensitive personal data protection (requests will be evaluated according to the GDPR—Reg. UE 2016/679 and its ethical guidelines).

## Abbreviations

The following abbreviations are used in this manuscript:

fNIRS	Functional Near-Infrared Spectroscopy
Indstep	Individual step
Coopstep	Cooperation step
Agrstep	Agreement step
GDMS	General Decision-Making Style
MS	Maximization Scale
BFI-10	10-item Big Five Inventory
HHb	Deoxygenated hemoglobin
O2Hb	Oxygenated hemoglobin
EDi	Euclidean distance index
